# Adaptive Auricular Point Acupressure for Sleep Disturbance in Women with Breast Cancer: A Randomized Controlled Trial

**DOI:** 10.1155/2022/8637386

**Published:** 2022-10-31

**Authors:** Yuyan Wang, Xinyu Li, Xiaopeng Ji, Jing Wu, Junxin Li, Wei Zheng, Caiqin Wu, Lan Huang, Zhaohui Geng, Jie Zhou

**Affiliations:** ^1^School of Nursing, Shanghai University of Traditional Chinese Medicine, Shanghai, China; ^2^School of Nursing, University of Delaware, Newark, DE, USA; ^3^School of Nursing, Johns Hopkins University, Baltimore, MD, USA; ^4^Longhua Hospital, Shanghai University of Traditional Chinese Medicine, Shanghai, China

## Abstract

**Objectives:**

The study aimed to evaluate the preliminary effect and efficacy of auricular point acupressure (APA) on the quality of sleep in women with breast cancer who were undergoing chemotherapy. *Sample & Setting*. We conducted a randomized controlled trial on 68 patients with breast cancer who reported poor sleep quality based on the Pittsburgh Sleep Quality Index (PSQI) scores (>7). *Methods & Variables*. Participants were randomly assigned to an APA treatment group or a control group. Patients in the APA group had magnetic pellets attached to selected auricular points and were instructed to apply pressure to these points 4×/day for three consecutive weeks. We objectively measured sleep quality using the Actiwatch Spectrum and the PSQI at the baseline and postintervention. Statistical analyses of changes in sleep data were performed using the *t*-test, a rank-sum test, and analyses of covariance.

**Results:**

In patients treated with APA, the PSQI total score and sleep onset latency had significantly decreased, while the total sleep time and sleep efficiency had significantly increased. Although the total PSQI score differed between groups at the baseline, ANCOVA results showed that the APA group had a significantly lower total PSQI score.

**Conclusion:**

APA could be an inexpensive and effective approach to improving sleep quality and reducing sleep disturbance in patients with breast cancer. Further research needs a larger sample size to verify our findings.

## 1. Background

Sleep disturbances are pervasive health concerns among patients with breast cancer. More than 60% of patients with breast cancer have poor sleep quality, of whom 22% report difficulty falling asleep, 42% report being easily awakened, and 23% report daytime sleepiness [1]. These sleep disturbances, which are partly attributable to cancer treatment, are long lasting. A systematic review reported that during chemotherapy, patients with breast cancer tended to report more severe sleep disturbance and could experience sleep problems throughout the chemotherapy cycle [2]. Sleep disturbance has been associated with an increased risk of comorbid conditions, increased levels of depression and anxiety, daytime sleepiness and fatigue, and decreased quality of life (QoL). Therefore, advancing clinical care for patients with breast cancer with sleep disturbance is a critical component of facilitating recovery and improving QoL.

Previous work in this area has also focused on pharmacological treatments and cognitive-behavioral therapy (CBT). Hypnotics such as benzodiazepines are the most commonly used treatment recommended by the American College of Physicians guideline [3]. However, adverse drug reactions, dependency on hypnotics, and drug interactions are of great concern during chemotherapy [4]. Psychological interventions such as CBT [5], psychoeducational therapy [6], mind-body treatments [7], and yoga [8] have been widely tested and demonstrated to have positive effects in helping patients manage sleep symptoms. However, cost, time, the limited number of trained practitioners, and poor patient compliance were barriers to continuing one study [9]. A new approach is needed to reduce sleep disturbance. As new forms of treatment for sleep disorders, complementary and alternative medicine (CAM) is emerging [10]. Prior studies have demonstrated the effectiveness of lemon verbena [11] and auricular acupuncture [12] for treating insomnia. Auricular point acupressure may be promising nonpharmacological sleep intervention [13].

APA is a method of treating physical and psychosomatic dysfunctions by stimulating specific points in the ear [14]. Auricular stimulation is gentle fingertip pressure applied to a seed or pellet attached to the external part of the ear. APA is a well-established treatment strategy in traditional Chinese medicine (TCM) and has been employed for approximately 2500 years [15]. TCM views the ear as directly or indirectly connected with each of the internal organs and 12 meridians [16]. Since Dr. Paul Nogier introduced the inverted-fetus map of the outer ear in 1957, the theory has become so widespread that the entire body corresponds with parts of the ear [17]. Finally, APA is convenient, noninvasive, and cost effective, with few side effects [18].

Evidence has shown that auricular therapies hold great potential to improve sleep quality. According to reflex theory, APA can stimulate the brain to regulate pathological-reflex centers, alter levels of serum anti-inflammatory compounds, and induce reflex reactions in the body to relieve symptoms [19–22]. The nerve distribution in the aural region also affects the autonomic nervous system, thereby promoting sleep quality [19]. In addition, the mechanism of APA in sleep disturbance might involve the regulation of melatonin [23]. A systematic review of 15 randomized controlled trials (RCTs) that applied APA to participants with primary insomnia showed that APA was more likely to improve the clinical effective rate, sleep efficiency, and PSQI total score than control treatments (sham auricular acupuncture, placebo, or medications) [24]. Another systematic review of 40 RCTs integrated 16 studies of APA stimulation and found that auricular acupressure as monotherapy or combined with routine care was significantly more efficacious than routine care or no treatment [25]. However, the deficiency of rigor in trial design tends to obscure the real effect of APA on insomnia due to bias [26]. Furthermore, most outcome measurements, such as PSQI, sleep diaries, and effective rate, reflect patients' subjective experiences. Only a few studies used polysomnography in the appraisal of the effectiveness of auricular therapy, but portable and objective measurements are still lacking [25]. Additionally, a one-size-fits-all APA treatment protocol was given where patients received the same APA protocol in one study. Nonetheless, based on the experiences of TCM practitioners, the selection of auricular points must follow the principle of dialectical treatment.

Given the need to improve sleep quality among patients with breast cancer during chemotherapy, we decided to examine the effect of a 3-week APA treatment protocol on (a) objective sleep parameters using wrist actigraphy and (b) subjective sleep quality (SSQ) ratings using the PSQI.

## 2. Methods

### 2.1. Design

In this study, we adopted an RCT design to examine the effectiveness of APA in sleep disturbance in patients with breast cancer. Patients who experienced poor sleep quality during chemotherapy were randomized into two groups: an APA group, with magnetic pellets attached to designated auricular points for sleep and a control group, who received standard care. In addition, all patients received training on sleep hygiene. The treatment duration was 3 weeks. The study was reported according to the Consolidated Standards of Reporting Trials (CONSORT) checklist (S1 CONSORT checklist).

### 2.2. Participants and Setting

We recruited a convenience sample of 68 patients from the specialist breast unit of three grade A hospitals at the third class in Shanghai, China, from January 1, 2019, to January 30, 2020. Participants were recruited if they (1) were > 18 years old, (2) were patients with breast cancer undergoing adjuvant chemotherapy for the second or a subsequent cycle of treatment [18], (3) complained of sleep disturbance (difficulty initiating sleep for > 30 min, staying asleep, or awakening prematurely) ≥ 3 nights per week, or (4) reported a PSQI score of > 7 [27]. Individuals with inflammatory lesions, wounds, or allergy to tape on the ears and patients who had received APA in the 3 months prior to enrollment were excluded.

All patients were between their first and last chemotherapy cycles. Most of their chemotherapy regimens were a combination of Adriamycin/epirubicin, cyclophosphamide, and docetaxel. Typically, patients underwent 4–8 cycles of chemotherapy every 3 weeks.

### 2.3. Random Assignment and Blinding

Participants were allocated to the APA or control group at a 1 : 1 ratio via stratified block randomization according to their age, with a block size of four. We generated a table of random numbers using Microsoft Excel (Microsoft Corp., Redmond, WA, USA) and kept the random allocation sequence in opaque sealed envelopes. The data analyst was blinded to the participants' group assignments.

### 2.4. Control Intervention

In the control group, patients received verbal and written health education on sleep hygiene from the start. This training aimed to correct patients' common misunderstandings about sleep disturbances and to establish proper sleep practices. Patients were recommended that they develop a fixed bedtime/wake-up time schedule, engage in physical exercise, keep the sleep environment cool, quiet, and dark, avoid exciting TV shows or books before bedtime, and limit caffeine intake [28].

### 2.5. Adaptive Auricular Point Acupressure Intervention

In the APA group, participants received both sleep hygiene education and adaptive-APA treatment. After the systematic review and expert group meeting, we chose Shenmen (TF4), Xin (heart; CO15), and Pizhixia (subcortex; AT4) as the main points with which to treat sleep disturbance. We also identified points corresponding to patients' symptoms using systematic auricular strategies. Identification of acupoints was a three-step process: (1) Querying participants on symptoms they were experiencing besides sleep disturbance, (2) Visually inspecting the ear for any discoloration or deformity of the auricle, and (3) Using an electronic point finder to identify acupoints. Point selection was based on the Zang organ and meridian theory of TCM [15] and on reflex theory [20] (see Table 1). The number of points treated and their specific locations on the ears of each patient varied slightly because each patient experienced different symptoms in different areas of the body, which projected onto different ear points according to somatic topography. We identified 3–6 total ear points per participant and located them with the guidance of the Chinese Standard Ear-Acupoints Chart [19]. We secured a magnetic pellet (approximately 1.5 mm in diameter) to each acupoint with a small piece of adhesive tape (7 mm × 7 mm). Pellets and tape were attached to only one ear of each patient over 3 days; we alternated between ears to avoid local irritation or ulceration on auricular points. Participants were asked to press the pellets between the thumb and the forefinger 4×/day (i.e., morning, noon, evening, and 30 min before bedtime) for 3 min each time. We directed them to exert this pressure to the point of an obvious but tolerable feeling of pain, thus creating swelling and warmth at auricular points. APA treatment was conducted over a 3-week period, with two office visits for pellet replacement per week.

### 2.6. Ethical Considerations

This research was approved by the Medical Ethics Committee of Longhua Hospital affiliated with the Shanghai University of TCM (No. 2019LCSY059). We explained the study objectives and individuals' rights and risks to all patients. The study was initiated after participants agreed and signed informed consent forms.

### 2.7. Study Procedures

The researcher provided detailed trial information to these potential participants, obtained their signed consent, and collected baseline assessments. This baseline investigation was performed on the day participants were hospitalized for chemotherapy, which was 1 day prior to chemotherapy being administered. The primary outcome measurement, sleep status, was assessed using an Actiwatch Spectrum portable actigraphy device (Philips Respironics, Murrysville, PA, USA) and the PSQI. The secondary outcome was QoL. Participants wore Actiwatch, which measures sleep activity, on the wrist of the nondominant hand at the time of admission and removed it after 72 h. To assess sleep quality and quantity, we measured the total sleep time, sleep efficiency, sleep latency, and the number of awakenings during sleep. After baseline investigation, participants were randomized into either the adaptive APA group or control group using randomly generated numbers.

During the study, all participants received verbal and written sleep hygiene education as per standard care on every hospital admission. Patients in the APA group received APA intervention in a treatment room. We identified ear acupoints using both an electronic acupoint locator and systematic auricular strategies, which included inspection for visual abnormality and probing for tenderness. The indicator on the electronic point finder lights up and beeps when acupoints corresponding to particular target symptoms are probed. When the indicator lit up, we asked the participant to describe the symptom they were experiencing. After acupoints were identified, the researcher placed a magnetic pellet on each of the participant's acupoints and secured it using tape; this procedure took 5–10 min. The researcher demonstrated the technique for applying pressure to acupoints with the thumb and the index finger. Then, the participants were asked to perform the technique to verify that they understood it. Moderate APA stimulation as described above under “adaptive auricular point acupressure intervention” was used in this study. We also instructed the participants to alternate pellets and tape between their left and right ears twice a week. This minimized the risk of an allergic reaction to the tape and maintained or restored acupoint sensitivity prior to the next treatment.

### 2.8. Variables

#### 2.8.1. Objective Sleep Measures

Participants wore Actiwatch to measure their sleep parameters. Actiwatch is a valid, reliable device that detects and records wrist movements using a triaxial accelerometer; it records sleep-wake patterns [29]. Actiwatch counts were downloaded in minute-by-minute epochs. We instructed the participants to wear the watch on the nondominant hand for three consecutive days and, during this time, to avoid taking it off or obscuring the light sensor. We used the Actiware software to analyze the data recorded by using Actiwatch, which yielded the following sleep parameters:

  Bedtime (BT): Time when the subject went to bed with the intent to sleep
  Get up time (GUT). Time when the subject rose from bed for the final time
  Time in bed (TB). Period of time between BT and GUT
  Total sleep time (TST). Total time within rest intervals scored as sleep
  Onset latency (OL). Period of time between BT and sleep start time
  Sleep efficiency (SE). Percentage of time in bed actually spent sleeping
  Wake after sleep onset (WASO). Number of waking minutes between sleep start and sleep end times
  Awake. Number of continuous blocks of time awake

#### 2.8.2. Subjective Sleep Quality

We used the PSQI to evaluate patients' sleep quality during the previous month. The instrument consists of 19 items measuring seven components (i.e., SSQ, sleep latency, sleep duration, habitual sleep efficiency, sleep disturbances, use of sleeping medication, and daytime dysfunction) [30]. Each component is scored 0–3 points, with 0 indicating “no difficulty” and 3 indicating “severe difficulty.” A global PSQI score is obtained by the sum of its seven component scores, which range from 0 to 21. Higher scores mean worse sleep quality. In the Chinese population, a global score >7 represents poor sleep quality. The sensitivity and specificity of the PSQI are reported to be 98.3% and 90.2%, respectively [27].

#### 2.8.3. Quality of Life

Functional Assessment of Cancer Therapy-Breast (FACT-B): The QoL parameters were assessed using the Functional Assessment of Cancer Therapy (FACT) questionnaire with a specific module for patients with breast cancer. The fourth version of the FACT-B questionnaire consists of 36 items divided into four primary QoL domains: physical wellbeing, social/family wellbeing, emotional wellbeing, and functional wellbeing, plus a special subscale for patients with breast cancer [31]. FACT-B is a sound, cross culturally valid assessment of QoL.

Adverse events: during each visit, the participants receiving APA treatment were observed and asked about adverse events (e.g., broken skin, pain, numbness, itching, and irritation).

Demographic and disease-related data: in this study, we used the demographic questionnaire to collect the following patient information: age, marital status, educational level, work status, religion, cancer stage, chemotherapy cycle, and emotional state.

### 2.9. Data Analysis

We used descriptive statistics to summarize participants' demographic characteristics and disease-related data. Demographic and disease-related differences between the APA and control groups were examined using the *t*-test, *χ*^2^, or rank-sum test. We calculated the mean values of Actiwatch sleep parameters (TB, TST, OL, SE, WASO, and Awake), SSQ (PSQI), and QoL. A one-way analysis of covariance (ANCOVA) with a baseline measure as a covariate was used to compare changes in the PSQI between the two groups after intervention. We analyzed the differences in Actiwatch sleep parameters and QoL parameters between groups using the independent *t*-test, and the Mann–Whitney *U* test after intervention. Each analysis was performed at the significance level of *P* < 0.05. We analyzed all statistical data using SPSS software version 24.0 (IBM Corp., Armonk, NY, USA) for Windows.

## 3. Results

### 3.1. Participant Recruitment, Withdrawal, and Retention

**Figure 1:**
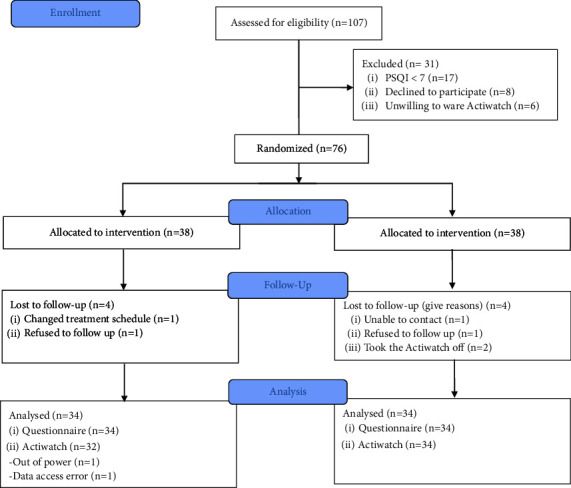
Flowchart of participant recruitment.

The process of participant recruitment is illustrated in Figure 1. One hundred seven patients contacted the researcher to express their interest in the study; of these, 76 patients were selected to participate in the study and scheduled for the initial assessment. We excluded participants from the study if they presented with a PSQI <7 (*n* = 17), were unwilling to wear Actiwatch (*n* = 6), and refused to participate in general (*n* = 8). During the study, eight participants were lost to follow-up due to changed treatment schedule (*n* = 1), refusal to follow up (*n* = 2), inability to contact (*n* = 1), and taking off Actiwatch to avoid monitoring (*n* = 4). Sixty-eight patients completed the trial, yielding a retention rate of 89.5% (both groups). Of these patients, two sets of Actiwatch data were missing due to a power outage and a data access error.

### 3.2. Demographic Characteristics and Disease-Related Data at Baseline

Of the total 68 participants recruited, the mean age was 53.47 years (standard deviation (SD), 11.88 years; range, 29–81 years). More than half (*n* = 39 (57.35%)) had education beyond high school. The majority were unemployed (*n* = 41 (60.29%)), married (*n* = 61 (89.71%)), and nonreligious (*n* = 53 (77.94%)). All participants were on a 3-week chemotherapy cycle, and the mean duration of the chemotherapy cycle was 3.68 (SD, 1.72). Moreover, the majority of the participants were being treated for stages I and II BC (stage I, *n* = 18 (26.47%); stage II, *n* = 36 (52.94%); stage III, *n* = 10 (14.71%); and stage IV, *n* = 4 (5.88%)). No statistically significant differences were noted between the two groups in terms of demographic characteristics or disease-related data (Table 2).

### 3.3. Objective Sleep Outcomes (Actiwatch)

Table 3 summarizes the measurements of sleep parameters as detected by using Actiwatch. Changes in these sleep parameters between the two groups. At the baseline, the participants in both groups had onset latency >30 min, sleep efficiency <85%, and awakening after sleep onset ≤55 min, indicating poor sleep characteristics. We noted no significant between-group differences in any Actiwatch sleep parameter before intervention. After intervention, the total sleep time and sleep efficiency increased, while onset latency significantly decreased in the APA group (*P* < 0.05).

### 3.4. Subjective Sleep Quality (PSQI)

As the results showed, the two groups differed to a statistically significant degree in the SSQ score at the baseline (*P*=0.012). Considering that participants' preintervention sleep quality could have affected APA treatment outcomes, we used ANCOVA with the baseline PSQI total score as the covariate to compare changes in the SSQ scores between the two groups after intervention. The between-group difference in the total PSQI score reached significance (*P* < 0.001) post-treatment, indicating that the SSQ in the APA group improved more than that in the control group. The APA group did not differ to a statistically significant degree from the control group in SSQ before and after intervention (*P*=0.878; Table 4). Significant differences also existed between the two groups on the detailed PSQI component postintervention, including sleep latency, sleep duration, sleep efficiency, sleep disturbances, and daytime dysfunction.

### 3.5. Quality of Life

QoL changes as shown by FACT-B questionnaire responses in both groups are presented in Table 5. These scores did not differ to a statistically significant degree at the baseline. After intervention, the FACT-B total score obtained significant between-group differences in the emotional-wellbeing and functional-wellbeing domains (both *P* < 0.05). Analysis of self-reported QoL data after 3-week APA intervention indicated that physical wellbeing significantly improved by 3.89 points (*P* < 0.001), social/family wellbeing by 1.03 points (*P* < 0.001), emotional wellbeing by 2.00 points (*P* < 0.001), functional wellbeing by 2.47 points (*P* < 0.001), and the BC subscale score by 2.03 points (*P* < 0.05) in the APA group.

### 3.6. Adverse Events in APA Treatment

In the course of APA intervention, two participants experienced local pressure ulcers on their ear points. After removal of pellets and application of entoiodine, the broken skin healed within 5 days. One participant reported pain and minor nausea when receiving pressure on the sympathetic acupoint for the first time. These symptoms gradually disappeared after the intensity of pressure on the pellet was reduced. Moreover, no patient dropped out of the study due to these adverse effects.

## 4. Discussion

Our study examined an APA program targeting sleep disturbance in women undergoing chemotherapy for BC. Before being applied in the clinic, CAM should be verified using stringent, evidence-based methodologies, which also help innovative therapies develop and gain popularity [32, 33]. In recent years, a considerable number of studies have shown that APA is effective in improving SSQ in different populations [34–38]. This study further showed that APA with magnetic pellets significantly improved sleep quality as evaluated by both SSQ and actigraphy in patients with breast cancer with sleep disturbance. Additionally, participants in the adaptive APA group had better QoL than those in the control group. In terms of group homogeneity, group differences mainly emerged in the total PSQI score, suggesting that the participants in the APA group perceived worse sleep disturbances than those in the control group. Despite the RCT design, the small sample size might have led to sampling errors. We used ANCOVA with a baseline measure as a covariate to control this difference in subsequent analysis [39].

This study identified sleep characteristics of patients with breast cancer with sleep disturbance. A severe sleep symptom burden was reported while patients were receiving chemotherapy. SSQ, sleep latency, and habitual sleep efficiency were the top three highest-scoring dimensions of the PSQI. The total PSQI score in the APA group before intervention was 11.59 ± 2.73, which was consistent with the results of a previous study of patients with breast cancer [40]. For sleep parameters, the best cutoff values for distinguishing the general population from cancer patients are 30 min for OL, 25 min for WASO, and 85% for SE [41]. Our objective sleep results confirmed that the participants experienced longer OL and WASO, lower SE, and more awake time than the general population [42]. These findings were similar to those of the study by Chaoul [43] reporting Actiwatch results of patients with breast cancer.

Compared with the control group, all PSQI dimensions improved markedly in the APA group after intervention, except for SSQ and use of sleep medication. According to a meta-analysis of the effects of auricular acupuncture with seed or pellet attachments on primary insomnia, the difference in efficacy between treatment and control groups was statistically significant but not entirely clinically relevant [24]. In the study by Kuo et al. [36], participants with PSQI scores >5 received APA treatment for 6 weeks; the control group received advice on sleep hygiene practices. After treatment, the global PSQI score of the APA group dramatically decreased by 8.9 points, while that of the control group increased by 0.5 points. However, the study by Kuo et al. [36] lacked robust objective measurements of sleep and therefore might have failed to accurately represent the efficacy of APA. Our study was more specific, and in this study, we also used actigraphy to further examine the stimulatory effect of APA. In order to control environmental factors, all Actiwatch data were collected at the hospital.

In the Actiwatch appraisal, outcomes, the total sleep time, and sleep efficiency increased, and onset latency decreased in the APA group compared with the control group after intervention. After auricular therapy, the mean onset latency decreased to 15 min, the total sleep time increased to 7 h 39 min, and the mean sleep efficiency rose to 85% in the intervention group. In contrast with our findings, the study by Yoon and Park [40] showed that 6 weeks of auricular therapy using Vaccaria seeds evidently improved SSQ, but no significant differences appeared in sleep parameters detected by using Fitbit Charge HR (FitBit Inc., San Francisco, CA, USA). In our APA treatment protocol, magnetic pellets replaced Vaccaria seeds to produce a magnetic effect and stimulation via manual pressure since Suen et al. reported that magnetic pellets provided better therapeutic effects than Semen Vaccariae or Junci Medulla [16]. This suggested that the effectiveness of auricular therapy in sleep improvement was due to the magnetic effect. Furthermore, a previous study showed that pressure applied to magnetic pellets yields better sleep improvement; participants' TST and SE increased, while their OL, minutes, awake, and Stage 1 sleep decreased after the application of these pellets with stimulation. Nonetheless, no significant differences in minutes, awake, and during sleep occurred in our study. Given that our study included patients with breast cancer undergoing chemotherapy, we believe chemotherapy was associated with poor sleep. Symptoms such as nausea, hot flashes, pain, and nocturia contributed to sleep disruption after chemotherapy [44].

The effects of APA on both subjective and objective sleep quality were better than the effects of sleep hygiene education alone. A possible explanation of these improvements might relate to the regulatory effect of APA stimulation on the production and activity of inflammatory cytokines, potentially leading to reversal of chemoresistance as well as to immune tolerance [45, 46].

However, sleep disturbance scores in our study remained in the clinically significant range (PSQI > 7) after intervention. All participants receiving chemotherapy were on a 3-week cycle. Considering their treatment plans and burdens, we implemented a 3-week treatment protocol. Our findings suggested that this 3-week APA regimen might be appropriate for patients with breast cancer receiving chemotherapy. However, further studies are needed to explore the appropriate parameters of APA treatment.

APA is a relatively safe treatment, with few reports of infection or local pain [47]. In this study, broken skin, pain, and minor nausea were reported as adverse effects. Vas et al. [48] reported that auricular implants caused pressure ulcers in 18 of 265 participants, which might have been due to excess pressure frequency and continual stimulation. In our study, two participants experienced pressure ulcers from having their ear points pressed 7–8×/day, although the protocol suggested 4×/day. In the study by Vas, pressure was applied to the implants 3×/day and maintained for 7 days until the next replacement [48]. Therefore, a rest period was necessary for the auricular skin to recover before the next treatment. Pain is one of the most common adverse effects of APA [47]. One participant in our study reported pain and nausea when receiving APA for the first time. Participants in other studies have also reported pain [49–51], which could be relieved by reducing the frequency and intensity of pressure on seeds [52]. Nausea was also reported in four studies [53, 54]. It might have been due to excessive stimulation of the sympathetic nerve fibers wrapped around the pinna, which led to gastrointestinal symptoms; therefore, nausea might be partially manageable by reducing the intensity of acupressure. No participants dropped out of our study due to these adverse effects, which implied that symptoms were tolerable. Future studies should emphasize the importance of informing participants about these possible adverse effects. Moreover, excessive stimulation and continual pressure should be avoided when implementing APA treatment. Magnetic pellets require additional safety precautions; patients with implanted pacemakers or electronic devices should avoid using magnetic pellet attachments [55].

### 4.1. Study Limitations

The current study has several limitations. One was the relatively small sample studied; future studies will require a larger number of participants to substantiate our findings. Another limitation is that the Hawthorne effect and the social desirability bias effect cannot be ignored in subjective appraisals. Monitoring objective sleep via actigraphy is necessary to evaluate the real effect of APA interventions. Further double-blinded studies with the placebo control are required to eliminate the subjective effects that existed in the current study. Moreover, a long-term follow-up study will be necessary to ascertain the long-term effects of APA. Failure to track participants' adherence was the final limitation. The effectiveness of APA depends specifically on compliance with the APA protocol (pressing ear points 4×/day for 3 min/time). Most participants did not regularly keep a track of APA practice. Nevertheless, only one participant in the control group could not be contacted, and participants in the APA group stated that they had adhered to the APA protocol during each outpatient visit or during telephone conversations.

## 5. Conclusion

The current study demonstrated that 3-week APA treatment using magnetic pellets effectively relieved both subjective and objective sleep disturbance. APA treatment is noninvasive, simple to operate, and inexpensive to apply. Based on its abovementioned effectiveness and advantages, we recommend the use of APA to deal with the common and distressing symptoms of sleep disturbance in patients with breast cancer. APA intervention is within the scope of nursing practice with TCM training. Although few side effects were reported, attention should still be paid to monitoring the performance of the APA protocol.

## Figures and Tables

**Table 1:** Auricular points selected for participants.

Auricular points		Rational for selection
Main points (used for each participant in the APA group)	Shenmen (TF4)	Promoting inhibition (tranquilizing) effects
	Xin (heart, CO15)	Calming the mind based on the Zang organ and meridian theory of TCM
	Pizhixia (subcortex, AT4)	Harmonizing excitement and inhibition of the cortex

Corresponding points (varied for each participant in the APA group)	Shen (kidney, CO10)	Based on reflex theory, alleviating symptoms such as nocturia, nausea and vomiting, anxiety, or pain.
	Wei (stomach, CO4)	
	Jiaogan (sympathetic, AH6a)	
	Location of body pain	

**Table 2:** Demographic characteristics and disease-related data between groups.

Variables	Groups			
	Control group (*n* = 34)	APA group (*n* = 34)	*t*/*X*^2^/*Z*	*p*
Age, mean (SD)	54.35 (12.84)	52.59 (10.50)	0.610^a^	0.544
BMI^#^, mean (SD) kg/m^2^	23.38 (2.74)	23.79 (2.79)	−0.619^a^	0.538

Education level, *n* (%)			0.287^b^	0.866
Middle school or below	15 (44.12%)	14 (41.18%)		
High school	9 (26.47%)	11 (32.35%)		
College or above	10 (29.41%)	9 (26.47%)		

Employment status, *n* (%)			0.061^b^	0.804
Employment	13 (38.24%)	14 (41.18%)		
Unemployment	21 (61.76%)	20 (58.82%)		

Marital status, *n* (%)			0.159^b^	0.690
Married	31 (91.18%)	30 (88.24%)		
Single/divorced/widow	3 (8.82%)	4 (11.76%)		

Religion, *n* (%)			0.219^b^	0.896
None or other	26 (76.47%)	27 (79.41%)		
Christian	3 (8.82%)	2 (5.88%)		
Buddhist	5 (14.71%)	5 (14.71%)		

Insomnia before BC, *n* (%)			0.159^b^	0.690
No	31 (91.18%)	30 (88.24%)		
Yes	3 (8.82%)	4 (11.76%)		

Cancer stage, *n* (%)			−0.148^b^	0.882
I	10 (29.41%)	8 (23.53%)		
II	16 (47.06%)	20 (58.82%)		
III	7 (20.59%)	3 (8.82%)		
IV	1 (2.94%)	3 (8.82%)		

Chemotherapy cycles received, mean (SD)	3.47 (1.62)	3.88 (1.82)	−1.017^c^	0.309
HADS_anxiety, mean (SD)	4.29 (3.74)	4.59 (3.14)	−0.685^c^	0.494
HADS_depression, mean (SD)	4.76 (2.90)	4.53 (2.67)	−0.179^c^	0.858

Abbreviation: APA, auricular point acupressure; BC, breast cancer; BMI, body mass index; HADS, Hospital Anxiety and Depression Scale. ^a^Independent *t*-test was used. ^b^Chi-square test was used. ^c^Mann–Whitney *U* test was used. The Chi-square test was used to examine the differences of cancer stage between two groups.

**Table 3:** Comparisons of the sleep parameters measured by Actiwatch between the two groups (mean (SD)).

Sleep parameters	Pretest		Post-test			
	Control group (*n* = 34)	APA group (*n* = 34)	Control group (*n* = 34)	APA group (*n* = 34)	*t*/*Z*	*P*
TB (hh : mm : ss)	9 : 13 : 33 (1 : 15 : 47)	8 : 42 : 07 (2 : 00 : 43)	8 : 17 : 42 (1 : 17 : 24)	8 : 55 : 53 (1 : 24 : 30)	−2.000^c^	0.045^∗^
TST (hh : mm : ss)	7 : 28 : 52 (1 : 10 : 16)	6 : 47 : 30 (1 : 44 : 53)	6 : 31 : 59 (1 : 13 : 01)	7 : 39 : 15 (1 : 30 : 24)	−3.356^a^	0.001^#^
OL (min)	30.19 (25.48)	33.33 (25.57)	29.69 (21.12)	15.97 (13.66)	−3.130^c^	0.002^#^
SE (%)	81.25 (7.01)	78.14 (9.26)	78.81 (6.56)	85.39 (5.77)	−4.350^a^	<0.001^#^
WASO (min)	55.90 (30.20)	55.91 (26.66)	52.91 (22.56)	43.39 (19.30)	1.854^a^	0.068
Awake (times)	46.14 (16.14)	41.95 (19.67)	41.87 (14.05)	38.36 (14.13)	1.020^a^	0.311

Abbreviation: APA, auricular point acupressure; OL, onset latency; SE, sleep efficiency; TB, time in bed; TST, total sleep time; WASO, wake after sleep onset. ^a^Independent *t*-test was used. ^c^Mann–Whitney *U* test was used. ^*∗*^*P* < 0.05,^#^*P* < 0.01.

**Table 4:** Comparison of mean PSQI scores between the two groups (mean (SD)).

	Groups			
	Control group (*n* = 34)	APA group (*n* = 34)	*t*/*F*	*p*
PSQI total score				
Pretest	9.97 (2.44)	11.59 (2.73)	−2.574^a^	0.012^∗^
Post-test	9.91 (2.31)	7.59 (2.44)	54.107^e^	<0.001^#^
*T*	0.155^d^	12.893^d^		
*P*	0.878	<0.001^#^		

Abbreviation: APA, auricular point acupressure; PSQI, Pittsburgh Sleep Quality Index. ^a^Independent *t*-test was used. ^d^Paired *t*-test was used. ^e^Covariance analysis was used. ^*∗*^*P* < 0.05,^#^*P* < 0.01.

**Table 5:** Comparisons of life quality scores between the two groups (mean (SD)).

Items of FACT-B	Pretest		Post-test		*t*/*Z*	*P*
	Control group	APA group	Control group	APA group		
Physical wellbeing	21.15 ± 4.47	21.03 ± 4.82	22.06 ± 3.20	22.82 ± 4.17	−1.69^c^	0.092
Social/family wellbeing	19.15 ± 4.07	18.35 ± 3.28	18.38 ± 4.34	19.38 ± 3.69	−1.02^a^	0.310
Emotional wellbeing	17.15 ± 5.09	17.32 ± 4.38	17.44 ± 3.74	19.32 ± 2.98	−2.11^c^	0.035^∗^
Functional wellbeing	15.65 ± 4.13	14.56 ± 4.14	14.82 ± 3.86	17.03 ± 4.19	−2.26^a^	0.027^∗^
Breast cancer subscale	23.85 ± 6.03	24.65 ± 5.29	24.24 ± 5.62	26.68 ± 4.48	−1.98^a^	0.052
FACT-B total score	96.94 ± 15.48	95.91 ± 16.85	96.94 ± 13.25	96.94 ± 13.25	−2.40^a^	0.019^∗^

Abbreviation: APA, auricular point acupressure; FACT-B: Functional Assessment of Cancer Therapy-Breast. ^*∗*^*P* < 0.05,^#^*P* < 0.01.

## Data Availability

The data used to support the findings of this study are available from the corresponding author upon request.

## Supplementary Materials

Supplementary Materials CONSORT 2010 checklist of RCT.
